# Baclofen Toxicity in a Dialysis-Dependent Patient: A Case Report

**DOI:** 10.7759/cureus.44932

**Published:** 2023-09-08

**Authors:** Asma Al Marzouqi, Abdullah M Al Alawi

**Affiliations:** 1 Internal Medicine, Residency Training Program, Oman Medical Specilaty Board, Muscat, OMN; 2 Medicine, Sultan Qaboos University Hospital, Muscat, OMN

**Keywords:** hemodialysis, muscle relaxant, chronic kidney disease, encephalopathy, baclofen toxicity

## Abstract

Baclofen toxicity is a significant concern, particularly in patients with advanced chronic kidney disease. We present a case of a 74-year-old female who developed depressed consciousness after receiving 20 mg of baclofen for back pain control. A presumptive diagnosis of baclofen toxicity was made, and the patient underwent continuous hemodialysis sessions, leading to neurological recovery within two days. This case highlights the risk of baclofen toxicity in dialysis-dependent patients with advanced chronic kidney disease, emphasizing the importance of vigilance and alternative treatment options in this population.

## Introduction

Baclofen is a lipophilic analog of gamma-aminobutyric acid that crosses the blood-brain barrier and acts as a muscle relaxant. 85% of the drug is excreted unchanged by the kidneys [1]. Therefore, the duration of action of baclofen is prolonged in patients with renal impairment. Most cases of baclofen toxicity among dialysis-dependent patients are reported two to three days up to 16 weeks after ingestion [2]. We report the case of a dialysis-dependent patient who had encephalopathy within 12 hours after ingesting 20 mg of baclofen.

## Case presentation

A 74-year-old female with end-stage renal disease undergoing regular hemodialysis (three sessions weekly) presented to the Emergency Department with an altered level of consciousness. She has a medical history of hypertension, dyslipidemia, and atrial fibrillation; however, she is not on anticoagulation therapy. Her daily medications are metoprolol (10 mg), amlodipine (10 mg), atorvastatin (20 mg), and aspirin (100 mg). Cognitively intact at baseline, she requires some assistance for daily activities and uses a walker for ambulation.

There was no preceding history of fever, gastroenteritis, jaundice, or trauma prior to her presentation. She had complaints of chronic back pain and was recently started on baclofen (10 mg). She consumed 20 mg over the span of 24 hours. Her family observed increased drowsiness and decreased responsiveness 12 hours after her second dose. No other sedative medication ingestion was reported.

**Figure 1:**
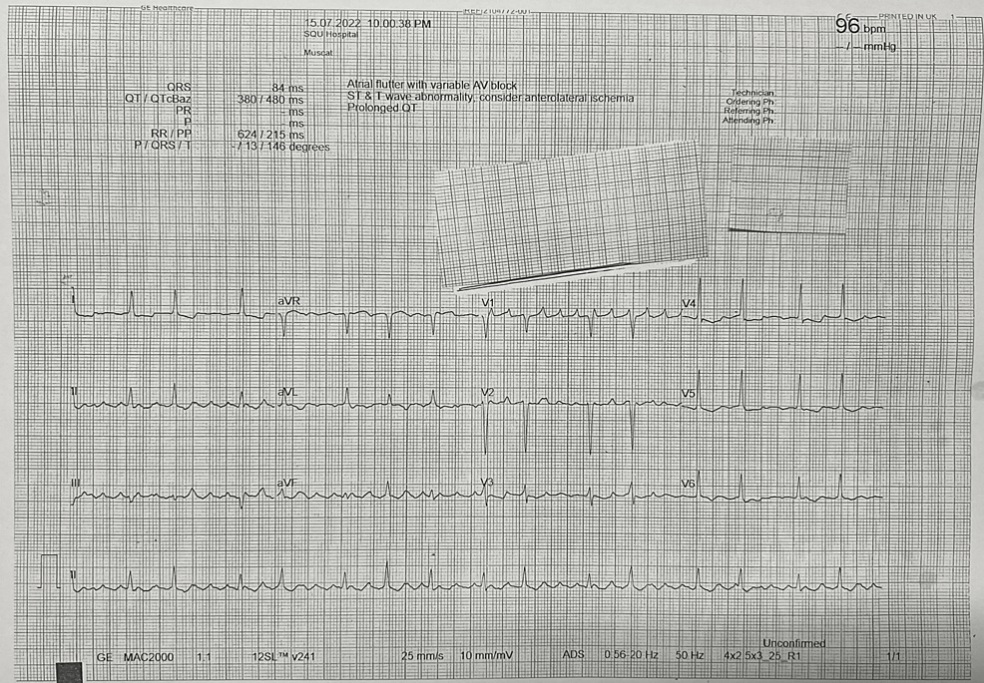
ECG shows atrial flutter with 2:1 block

On evaluation in the Emergency Department, the patient was obtunded, reacting only to deep painful stimuli, with a Glasgow Coma Scale score of E2V2M4. Pupillary examination showed equal size and reactivity to light. There was no neck rigidity or focal neurological signs. All other vital signs were within normal limits. A urine dipstick test was unremarkable. Laboratory investigations revealed: blood glucose - 150 mg/dL, serum sodium - 135 meq/L, serum potassium - 4.2 meq/L, serum calcium - 8.7 mg/dL, blood urea - 87 mg/dL, and serum creatinine - 4.4 mg/dL. A computed tomography (CT) scan of the head did not demonstrate any abnormalities. An electrocardiogram (ECG) displayed atrial flutter with a 2:1 block (Figure 1).

Further investigations, including full blood count, liver function tests and a bone profile-reactive protein, were unremarkable. Considering her medical history, clinical examination, laboratory results, and particularly her recent baclofen ingestion followed by altered sensorium, baclofen toxicity was strongly suspected. Other potential diagnoses, such as urinary tract infection and meningoencephalitis, were ruled out.

Despite undergoing a four-hour clearance dialysis, the patient's sensorium remained unchanged. Subsequently, consultations with a toxicologist and nephrologist were sought. They recommended continuous venovenous hemodialysis (CVVHD). She was then transferred to the intensive care unit (ICU), where a temporary dialysis line was placed, and CVVHD was initiated. This intervention led to a complete restoration of her sensorium within two days. By the third day, she had significantly improved and was discharged home in stable condition.

## Discussion

Baclofen is a central muscle relaxant used to manage spasticity, persistent muscle contractions, and intractable hiccups [1]. In managing spasticity, the recommended starting dose is 5 mg three times a day with a maximum dose of 80mg in a 24-hour period. The maximum recommended dosage for hiccups is 10-20 mg thrice daily to a maximum not exceeding 60mg in 24 [1-3]. Multiple factors can increase the risk of baclofen toxicity, including uremia, aging, and concomitant central nervous system pathology [2]. Two hours after baclofen ingestion, its serum level peaks, with a half-life of approximately 3.5 hours (range: 2.5-6.8 hours) in healthy subjects [4], and it is cleared by the kidney; however, in patients with renal failure, the half-life of the drug is prolonged [2,3]. In addition, baclofen can cross the brain-blood barrier as it is moderately lipophilic, with a cerebrospinal fluid (CSF) concentration 8.4 times less than plasma. As a result, the drug gets sequestrated in the brain of patients with chronic kidney disease (CKD), despite the blood level being in the therapeutic range of 80-400 ng/mL only.

Vlavonou et al. studied the pharmacokinetics (PK) of baclofen and found that its PK is significantly affected by kidney disease; their study was conducted on patients with mild (50-80 mL/min), moderate (30-50 mL/min), and severe (<30 mL/min) renal failure and dose reduction by one-third, half, two-thirds were suggested for mild, moderate, and severe renal failure, respectively [1,5].

The signs and symptoms of baclofen toxicity include central nervous system depression, ranging from drowsiness, lethargy, somnolence, and confusion to delirium coma. Paradoxically, myoclonus and convulsions may also occur due to disinhibition [1,6]. Brainstem reflexes may also be lost entirely, mimicking brain death [6]. A previous study has described baclofen intoxication as a “fun drug” that has been abused by adolescents seeking intoxication, which results in seizure and ICU admission for days and respiratory failure requiring mechanical ventilation [7]. Another study also found that baclofen levels obtained shortly after overdose correlated with the length of mechanical ventilation [8].

Approximately 55 cases of baclofen toxicity have been reported in patients with CKD, mainly among dialysis-dependent patients. El-Husseini et al. reviewed 41 patients with baclofen toxicity and found that the majority were aged >60 years (62.5%), men (56.3%), and on dialysis (62.9%); their presentations were almost always neurotoxicity when administered a range of 5-60 mg of the daily dose of baclofen with a mean dose of 20 mg from as early as two to three days to as long as 16 weeks after initiating the drug [2]. Khazneh et al. reported the case of a female patient with a sudden onset of altered mental status and unconsciousness state after ingesting a single tablet of 25 mg of baclofen for 12 hours, where she required five sessions of hemodialysis for a full recovery [9]. One study reported that baclofen toxicity develops following a single dose of 10 mg [1]. Hadjiyannacos et al. suggested that a safe dose in patients undergoing dialysis should not exceed 5 mg/day with careful monitoring during drug administration [10].

Our patient manifested encephalopathy following the consumption of two doses of baclofen, totaling 20 mg. There was a 24-hour gap between the doses, and the onset of encephalopathy symptoms occurred 12 hours after the last dose, during the interdialytic period. This led to a cumulative intake of 20 mg. To date, there have been no reports of toxicity associated with doses of baclofen less than 10 mg.

Recovery from baclofen toxicity can be accelerated by discontinuing the drug and enhancing its clearance by hemodialysis. Baclofen is effectively dialyzable because of its low molecular weight (213 Da), low volume distribution (0.7 L/kg, and low plasma protein binding (30%) [11]. In a study of the PK of baclofen, Wu et al. found that four hours of hemodialysis can shorten the half-life of baclofen from 15.5 hours to 2.06 hours and achieve a drug clearance rate of 79% [1,12]. Drug sequestration in the CSF is the mechanism of encephalopathy in patients undergoing regular dialysis; hence, repeated sessions may be required for drug clearance. Also, CVVHD offers continuous removal of toxins over extended periods, enhancing the clearance of drugs with large volumes of distribution, like baclofen, compared to intermittent HD. Continuous ambulatory peritoneal dialysis has also been applied as a successful treatment modality, with efficacy similar to intermittent hemodialysis [13]. Roberts et al. published an algorithm to approach patients with baclofen toxicity, suggesting supportive therapy in patients with mild toxicity and dialysis therapy for those with impaired renal function and severe symptoms [14].

## Conclusions

Baclofen toxicity is a serious condition, particularly in patients with renal impairment. Our case emphasizes that baclofen as low as a single dose of 10 mg and a cumulative dose of 20 mg can prove dangerous to dialysis-dependent patients, which can result in encephalopathy and require intensive care admission; hence, it is crucial to avoid baclofen in patients with CKD and look for alternative drugs.
